# How the first years of motherhood impact the cardiac autonomic profile of female healthcare professionals: a study by heart rate variability analysis

**DOI:** 10.1038/s41598-021-87596-y

**Published:** 2021-04-14

**Authors:** Laura Adelaide Dalla Vecchia, Beatrice De Maria, Giuseppina Cassetti, Letizia Clementi, Valeria De Grazia, Francesca Perego, Alberto Porta

**Affiliations:** 1 IRCCS Istituti Clinici Scientifici Maugeri, Via Camaldoli 64, 20138 Milan, Italy; 2 Department of Electronics Information and Bioengineering, Politecnico di Milano, Milan, Italy; 3 Department of Biomedical Sciences for Health, University of Milan, Milan, Italy; 4 Department of Cardiothoracic, Vascular Anesthesia and Intensive Care, IRCCS Policlinico San Donato, San Donato Milanese, Milan, Italy

**Keywords:** Physiology, Cardiology, Health occupations, Risk factors

## Abstract

The conciliation between career and family is a relevant issue for working women, in particular during the first years of motherhood. Data about the state of the cardiac autonomic regulation in working women with preschoolers are lacking. Aim of this study was to compare the cardiac autonomic profile of female healthcare professionals with and without preschoolers via the analysis of the variability of the time distance between two consecutive R-wave peaks (RR) from standard 24-h Holter electrocardiogram (ECG). Fifty healthy active female healthcare professionals were enrolled: 25 with at least one preschooler (W_KID) and 25 without (W_NOKID). A standard Holter ECG was obtained during a regular working day. Segments of 5000 consecutive RRs were selected during daytime (DAY) and nighttime (NIGHT). Heart rate variability analysis was performed and the following parameters were considered for comparison between the two groups: mean (μ_RR_), variance (σ^2^_RR_), and the absolute power in high frequency component (HF) of RR (HF_RR_) series. HF_RR_ was considered as a marker of vagal cardiac modulation. Only µ_RR_ significantly increased from DAY to NIGHT in both groups (699 ± 88 vs 887 ± 140 ms in W_KID and 728 ± 90 vs 942 ± 166 ms in W_NOKID). Instead, σ^2^_RR_ and HF_RR_ increased from DAY to NIGHT only in W_NOKID (from 3334 ± 2153 to 4816 ± 4063 ms^2^ and from 356 ± 334 to 1397 ± 1629 ms^2^, respectively). W_KID showed lower σ^2^_RR_ and HF_RR_ during NIGHT, compared to W_NOKID (2336 ± 3170 vs 4816 ± 4063 ms^2^ and 556 ± 950 vs 1397 ± 1629 ms^2^, respectively). The perceived stress according to the visual analogue scale was similar in the two groups (4.7 ± 2.1 in W_KID, 5.7 ± 2.1 in W_NOKID). The presence of preschoolers lowered nocturnal cardiac vagal modulation in female healthcare professionals. This might represent an adaptation with a finalistic purpose, scilicet the facilitation of a prompt reaction in case of a child’s need.

## Introduction

The conciliation between career and family is a relevant issue. Women are often sandwiched between more and more competitive and demanding working activities and traditional family duties. Debate exists about the impact of the multiple-role engagement, i.e. family and work roles, on psychological stress and quality of life. The “Depletion Hypothesis” considers the multiple-role engagement as a factor contributing to overload and strain, while the “Enrichment Hypothesis” as a factor enhancing individual’s resources, social connections, power, prestige and emotional gratification^1,2^. In any case, there might be a potentially stressful condition that, in turn, might influence women’s health status.

Independently from the family engagement, the stress induced by the working load has been associated to an increased risk for cardiovascular diseases development, together with other risk factors, such as physical inactivity, smoking, poor diet habits, overweight, hypertension, dyslipidemia, and diabetes^3–6^. This increased risk could be related to an altered cardiac neural regulation due to a reduced vagal and/or an augmented sympathetic modulation to the heart. The sympathovagal imbalance would entail a diminished adaptive capability of the cardiac control to respond to external perturbations^5^.

The cardiac autonomic profile (CAP) can be studied by means of the heart rate variability (HRV), i.e. beat-to-beat variation of the cardiac cycle defined as the time interval between two successive R-wave peaks (RR) from the electrocardiogram (ECG). HRV analysis allows to infer vagal modulation directed to the sinus node. Indications about the state of sympathetic modulation can be deduced only under the hypothesis that sympatho-vagal balance is working^7,8^. These indices vary physiologically in response to a number of daily life challenges, such as orthostatism and physical activity, denoting the transitional adjustment of the system with a finalistic purpose^9–13^.

The same indices assume prognostic significance when modified as the result of pathological conditions. For instance, decreased overall HRV, increased sympathetic and/or decreased vagal activity directed to the heart after a myocardial infarction, in hypertension and heart failure are related to increased mortality and morbidity^14–17^. On the other hand, therapeutic interventions that improve the sympathovagal balance may positively affect prognosis^18,19^.

In addition, HRV studies have demonstrated that also pregnancy and menopause influence the CAP^20–24^. During pregnancy, a high sympathetic modulation directed to heart and vessels has been found in women at the late stage of pregnancy compared to the early stage^25^. After menopause, an overall decreased HRV and a cardiac neural sympathetic predominance have been described^23^. In these conditions it is still unclear whether the modified CAP is expression of a physiological adaptation or of an increased risk of unfavorable events.

Maternity also represents a delicate and intense period of life, in terms of both physical and mental engagement, in particular in the first years. In fact, it is known that anxiety and depression easily develop^26^ with possible repercussions on both mothers, offspring and their future health status^26,27^. In this perspective, the study of the CAP in this setting might add important information in understanding physiological and pathophysiological mechanisms. We hypothesize that the CAP of working women might be influenced by the strain determined by the double burden, i.e. workload and child care.

To test this hypothesis, we compared the CAP of two groups of female healthcare professionals^28^, one group with at least one preschooler, and a second group without. HRV from a standard 24-h ECG recording provided the indices of the daily and nocturnal CAP. As it is recognized that stress could influence the state of the CAP^29^, we also evaluated the degree of the perceived stress in the study population.

## Methods

### Population

The study population included 50 healthy women: 25 with at least one preschooler (W_KID) and 25 women without (W_NOKID). The sample was of convenience. They all were full-time active healthcare professionals working at IRCCS Istituti Clinici Scientifici Maugeri in Milan, Italy. Sample size calculation was performed over preliminary data collected on a subgroup of W_KID (9) and W_NOKID (8) on the RR variance parameter assuming a power of 0.90 and a level of significance of 0.05. Inclusion criteria were: (1) fertile age between 25 and 45 years; (2) absence of ECG abnormalities. Exclusion criteria were: (1) part-time workers or on maternity leave; (2) any history of cardiovascular, respiratory or metabolic disease; (3) any current pharmacological therapy known to influence the CAP; (4) any ongoing acute disease; (5) alcohol consumption > 24 g/day (> 250 ml of wine, > 660 ml of beer, > 80 ml of spirits)^30^; (6) moderate to heavy smoking (> 8 cigarettes daily)^31^. Criteria were verified at a pre-enrolment screening visit. The standard 12-lead ECG was acquired using a standard ECG equipment (Eli 250, Mortara Instrument, USA).

The protocol adhered to the principles of the Declaration of Helsinki and was approved by the local ethics committee “CE ICS Maugeri-IRCCS, Pavia” (2131CE). Each enrolled subject signed a written informed consent before participating in the experimental protocol.

### Experimental protocol

At enrollment, each subject filled out a detailed questionnaire to collect information on demographics (date and place of birth, professional role, working experience), clinical history (smoking and alcoholic habits, menstrual cycle information, use of medications, previous or current medical problems), working schedules, social (type and hours of social activities per week), exercise (type and hours of physical exercise per week), and sleeping (hours of sleep per night, number and length of nocturnal awakenings) habits. A complete physical examination of all districts, checks of height, weight and arterial blood pressure via standard manual sphygmomanometer were performed.

At the beginning of a regular working day, avoiding the 3 days after a night shift and the 7 days after the first day of the menstrual cycle, a 3-lead 24-h ECG (360° eMotion FAROS, Mega Electronics, Finland; Sylco srl, Monza, Italy) was positioned. We asked the participants to avoid alcoholic and caffeinated beverages, as well as heavy physical activity, in the 24 hours preceding and during the ECG recording. The electrodes were adequately positioned to minimize noise. The sampling rate was 500 Hz. Participants were asked to go to bed by midnight and take diary of all activities. Before starting the recording, each subject rated the perceived stress on a Visual Analogue Scale (VAS), that consisted of an unmarked ruler with endpoints labelled as “no perceived stress” (0) and “very high perceived stress” (10)^32,33^. The scale therefore yielded a subjective score on perceived degree of stress between 0 and 10.

### Time series extraction

The modified lead II was chosen to favor R wave identification. From the ECG signal, RR interval time series were derived by an automatic algorithm. RR interval was defined as the temporal distance between two consecutive R-wave peaks. Automatic detection of R-wave peaks was manually checked to avoid missing beats or errors. In presence of premature beats or artifacts, correction by means of cubic spline interpolation was implemented. The fraction of corrections never exceeded 5% of the total considered samples. Segments of 5000 consecutive RR intervals were selected during daytime (DAY, from 1 to 5 p.m.) and during nighttime (NIGHT, from 1 to 4 a.m.) for further analyses. On these selections, an iterated analysis on windows of 250 consecutive RR intervals, with superposition of 200, was performed. All the time and spectral indices were calculated over each time window and the median of the whole distribution was taken as representative^34^. After linear detrending, mean (μ_RR_) and variance (σ^2^_RR_) of RR series were calculated, and expressed in ms and ms^2^, respectively.

### Power spectral analysis

Parametric power spectral analysis was performed on RR series described by an autoregressive model. The model order was chosen according to Akaike information criterion and power spectral density was decomposed into spectral components characterized by a central frequency. Each frequency component was labeled as high frequency (HF) component if its central frequency dropped in the HF (0.15–0.4 Hz) band^7^. The absolute power in HF band of the RR series, HF_RR_, defined as the sum of power spectral components classified as HF and expressed in ms^2^, was considered as an index of the vagal modulation directed to the heart^8,35^.

### Statistical analysis

Categorical data were reported as absolute number (percentage) and continuous data as mean ± standard deviation. The differences between W_KID and W_NOKID in continuous variables were tested by two-tailed Student *t* test in case of normal distribution (tested by means of Kolmogorov–Smirnov test) or Mann–Whitney rank test in case of non-normal distribution, while for categorical variables χ^2^ test was applied. Two-way repeated measures analysis of variance (one factor repetition, Holm-Sidak test for multiple comparisons) was performed to check the differences between the two groups (i.e. W_KID and W_NOKID) within the same experimental conditions (i.e. DAY and NIGHT) and the differences between experimental conditions within the same group. Pearson and Spearman correlation analyses were carried out to assess the correlation between CAP indices and the perceived degree of stress and the children’s age. Correlation analysis was carried out separately during DAY and NIGHT over the single groups (i.e. W_NOKID and W_KID) and pooling the data together regardless of groups and experimental condition. A *p* < 0.05 was always considered significant. Statistical analyses were carried out using Sigmaplot, Systat Software, Inc., Chicago, IL, version 11.0.

## Results

All subjects completed the experimental protocol and referred a regular night of sleep during the Holter ECG recording. Awakenings were recorded on the personal diary. Eight W_NOKID and 10 W_KID reported 1 to 2 nocturnal awakenings. These periods were not considered for the analysis. An adequate period for HRV analysis was available in all subjects. The demographic and clinical features of the enrolled population are shown in Table 1. Ten women were on birth control pills (6 in W_NOKID and 4 in W_KID). No other regular medications were declared. As regards W_KID, an only child was present in 48% of cases, 2 children in 52% of cases. The mean age of children was 33.6 ± 18.1 months. The number of holidays and shifts was similar for W_KID and for W_NOKID. The percentage of subjects performing regular exercise (at least 1 day/week for at least 4 weeks) was also homogenous in the two groups, as well as the amount of sleeping hours per night.

**Table 1:** Demographic and clinical features of the enrolled population.

	W_NOKID (n = 25)	W_KID (n = 25)	*p*	Cohen’s *d*
Age, years	35.4 ± 7.2	37.7 ± 5.6	0.220	0.357
BMI (kg/m^2^)	22.7 ± 3.7	23.4 ± 3.1	0.496	0.205
SAP (mmHg)	108 ± 11	109 ± 13	0.893	0.083
DAP (mmHg)	67 ± 10	70 ± 10	0.459	0.300
Sleep per night (h)	6.6 ± 0.9	6.4 ± 1.3	0.472	0.179
Smoking, n (%)	7 (28)	5 (20)	0.741	0.094
Regular physical exercise, n (%)	12 (48)	8 (32)	0.386	0.247
Physical exercise (h/week)	3.0 ± 1.6	2.7 ± 1.9	0.604	0.171
Regular social activities, n (%)	23 (92)	17 (68)	0.077	0.516
Social activities (h/week)	7.4 ± 8.0	3.3 ± 2.3	0.060	0.696
Employment			0.212	0.629
Nurse, n (%)	7 (28)	10 (40)		
Physician, n (%)	4 (16)	6 (24)		
Physiotherapist, n (%)	13 (52)	6 (24)		
Nursing assistant, n (%)	1 (4)	3 (12)		
Working hours (h/day)	7.9 ± 1.0	7.7 ± 1.4	0.174	0.164
Working experience (years)	10.9 ± 5.7	12.6 ± 4.5	0.280	0.332

W_NOKID, women without preschoolers; W_KID, women with at least one preschooler; BMI, body mass index; SAP, systolic arterial pressure; DAP, diastolic arterial pressure; *p*, type I error probability; Cohen’s *d*, Cohen’s standardized mean difference between groups. Data are presented as mean ± standard deviation or number (percentage).

**Figure 1:**
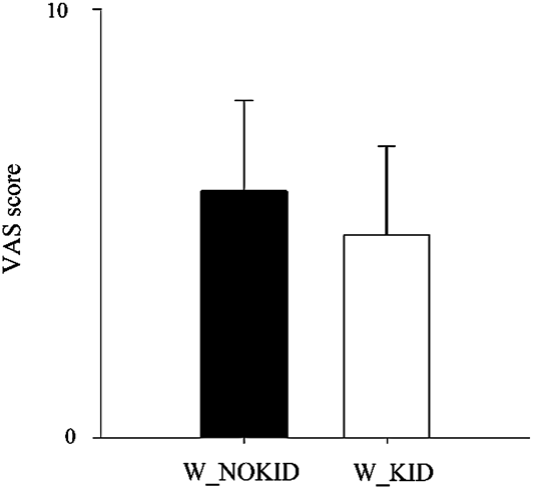
Results of perceived stress evaluation according to the Visual Analogue Scale (VAS) in W_NOKID (dark bar) and W_KID (white bar). Results are presented as mean ± standard deviation.

The perceived stress according to the VAS was surprisingly similar in the two groups (W_KID had 4.7 ± 2.1 and W_NOKID 5.7 ± 2.1, p = 0.087), as shown in Fig. 1.

**Figure 2:**
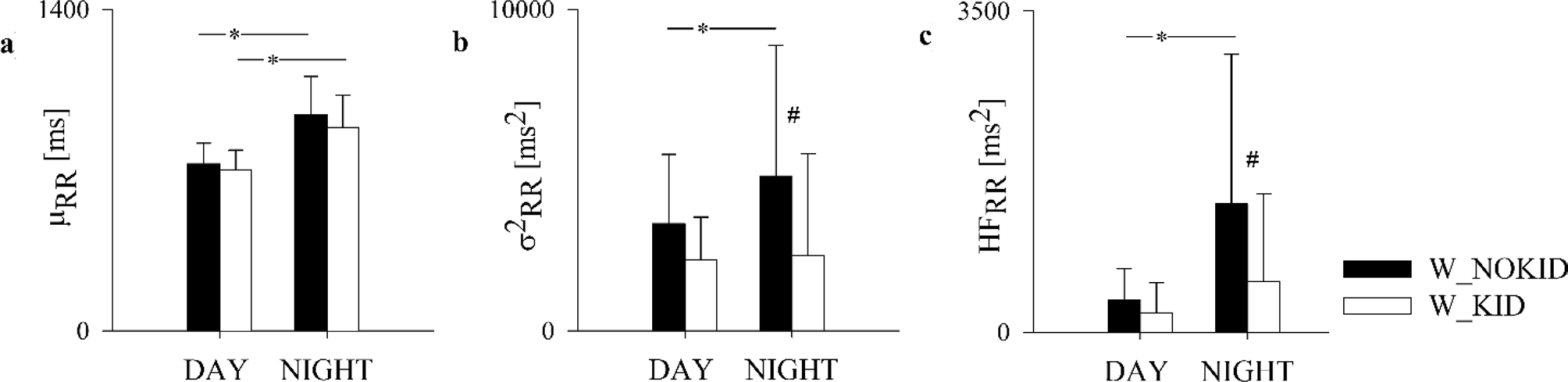
Results of power spectral analysis of heart rate variability during DAY and NIGHT in W_NOKID group (dark bars) and W_KID group (white bars). The indices μ_RR_ (**a**), σ^2^_RR_ (**b**), and HF_RR_ (**c**) are shown. Results are presented as mean ± standard deviation. The symbol * indicates *p* < 0.05 DAY vs NIGHT. The symbol # indicates *p* < 0.05 W_KID vs W_NOKID.

The results of the HRV analysis did differ as shown in Fig. 2. Only µ_RR_ significantly increased from DAY to NIGHT in both W_KID and W_NOKID (Fig. 2a), that is, as expected, healthy subjects are more bradycardic during the night. Instead, the expected increase of σ^2^_RR_ and HF_RR_ from DAY to NIGHT was observed only in W_NOKID. Thus, compared to W_NOKID, W_KID showed lower σ^2^_RR_ and HF_RR_ during NIGHT (Fig. 2b,c). In the W_KID group, the presence of one or two children did not differentiate the two small subgroups (12 vs 13 women).

**Figure 3:**
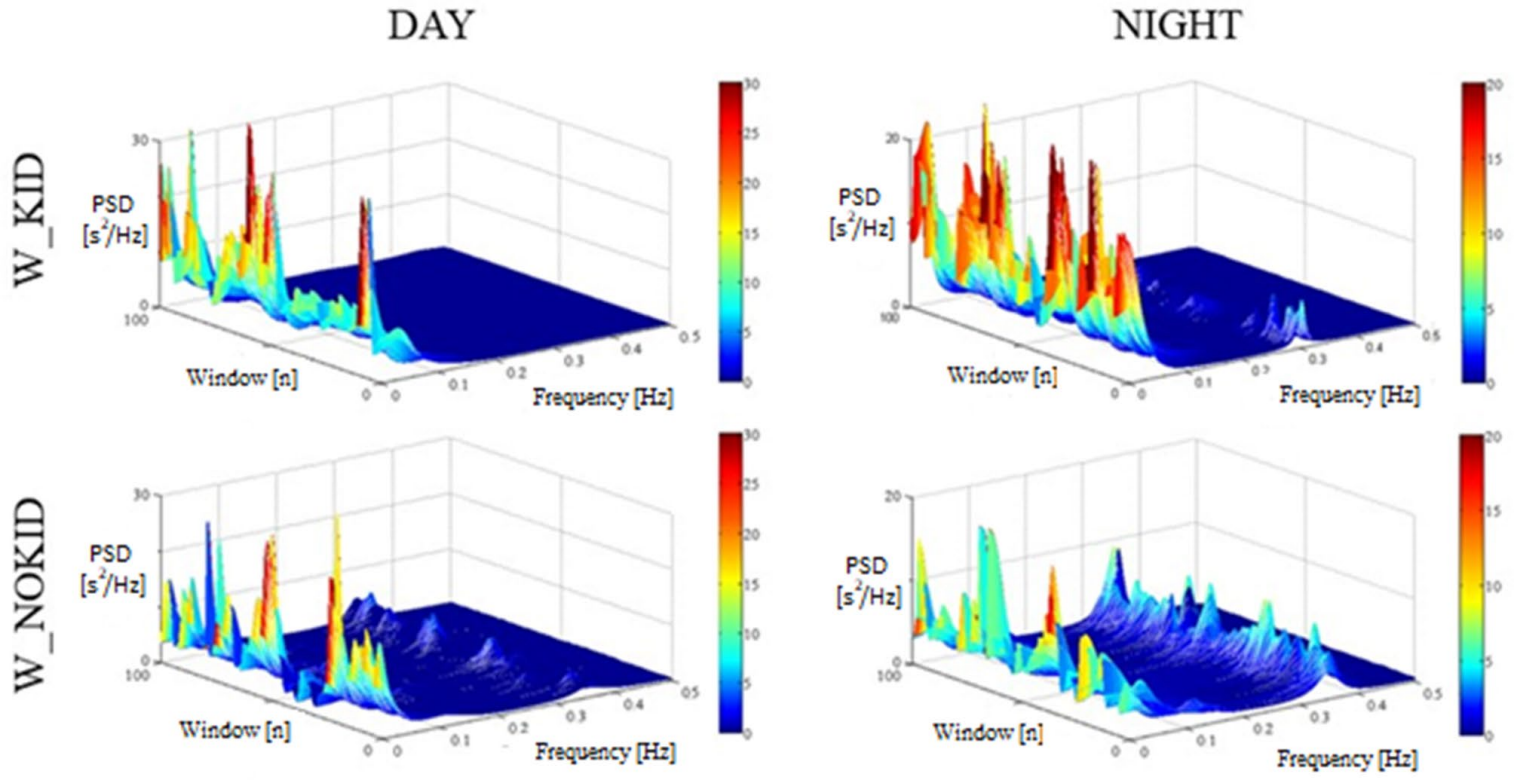
Illustrative examples of power spectral density of RR variability during DAY (left panels) and during NIGHT (right panels) computed in one subject of the W_KID group (top panels) and in one subject of W_NOKID group (bottom panels). Each 3D graph shows the power spectral density (PSD, z-axis) of the RR series as a function of the progressive frame of analysis (window, y-axis) and frequency (x-axis).

Figure 3 shows two representative examples of the power spectrum of the RR series during daytime (left side) and during nighttime (right side) in a 30-year-old female physician with a 2-year-old child (top panel) and in a 28-year-old nulliparous physician (bottom panel). Each 3D graph represents the power spectral density of the RR series (z-axis) for the considered frequency (x-axis), associated to each window of 250 consecutive RR intervals (y-axis). Of notice, during the night, the relative absence of peaks in the HF band in the woman with a child compared to the higher activity in the HF band of the nulliparous woman emphasizes the paradoxical modulation directed to the heart in presence of offspring.

Table 2 summarizes the results of the correlation analysis between HRV indices and the level of perceived stress assessed by the VAS. No significant correlations were found nor considering W_KID and W_NOKID separately, nor pooling the data together regardless of groups and experimental conditions. Thus, CAP modifications seemed to be unrelated to the degree of perceived stress.

**Table 2:** Results of the correlation between heart rate variability indices and VAS scale of perceived stress.

	W_NOKID				W_KID			
	r	p	ρ	p	r	p	ρ	p
μ_RR_ during DAY (ms)	− 0.336	0.101	0.220	0.287	− 0.009	0.965	0.033	0.873
σ^2^_RR_ during DAY (ms^2^)	− 0.089	0.670	0.075	0.717	0.001	0.999	− 0.063	0.761
HF_RR_ during DAY (ms^2^)	− 0.028	0.894	0.066	0.750	− 0.062	0.768	0.032	0.876
μ_RR_ during NIGHT (ms)	− 0.139	0.508	− 0.197	0.341	0.171	0.413	0.220	0.287
σ^2^_RR_ during NIGHT (ms^2^)	− 0.053	0.801	− 0.028	0.890	− 0.154	0.464	0.075	0.717
HF_RR_ during NIGHT (ms^2^)	− 0.014	0.946	− 0.096	0.644	− 0.166	0.428	0.067	0.750

W_NO KID, women without preschoolers; W_KID, women with preschoolers; RR, RR interval; μ_RR_, mean of RR; σ^2^_RR_, RR variance; HF_RR_, absolute power in high frequency band of the RR; DAY, daytime; NIGHT, nighttime; r, Pearson product moment correlation coefficient; ρ, Spearman rank correlation coefficient; p, type I error probability. Data are presented as mean ± standard deviation.

Also the correlation analysis between HRV indices and children’s age showed no statistically significant results (Table 3).

**Table 3:** Results of the correlation between heart rate variability indices and children’s age.

	r	p	ρ	p
μ_RR_ during DAY (ms)	0.098	0.642	0.104	0.615
σ^2^_RR_ during DAY (ms^2^)	− 0.197	0.345	− 0.200	0.334
HF_RR_ during DAY (ms^2^)	− 0.072	0.732	− 0.259	0.334
μ_RR_ during NIGHT (ms)	− 0.065	0.759	− 0.048	0.815
σ^2^_RR_ during NIGHT (ms^2^)	− 0.280	0.175	− 0.277	0.178
HF_RR_ during NIGHT (ms^2^)	− 0.219	0.293	− 0.228	0.270

DAY, daytime; NIGHT, nighttime; μ_RR_, mean of RR; σ^2^_RR_, RR variance; HF_RR_, absolute power in high frequency band of the RR series; r, Pearson product moment correlation coefficient; ρ, Spearman rank correlation coefficient; p, type I error probability.

## Discussion

In the present study, we demonstrated that the cardiac autonomic profile of a group of female healthcare professionals with preschoolers is different compared to that of a matched group of working women without children. During nighttime a decreased vagal modulation was demonstrated in the former group. However, such autonomic nervous system changes seemed to be unrelated to stress.

The enrolled subjects in the two groups were similar in respect of demographic and clinical parameters, life and working habits, and they were all healthy. The perceived stress was also similar in the two groups, as witnessed by the VAS scores. This finding was somehow surprising, as based on common experience and literature, women with double burdens (child care and workload) would be expected to define themselves as more stressed^36,37^. This seems to support the enrichment hypothesis^1,2^, according to which women who are involved in multiple social roles experience satisfaction rather than stress.

Thus, the presence of a commitment to childcare, rather than stress per se, would influence the CAP of the working women of this study.

In healthy subjects, the sympathovagal balance shifts towards an active mode^38^, instead the sleep is used as a period of cardiovascular relaxation and autonomic quiescence^32^.

In this study, heart rate physiologically decreased during nighttime in both groups, as mirrored by the µ_RR_ increase. Instead, the expected physiological increase of the vagal cardiac activity during the night, mirrored by higher σ^2^_RR_ and higher HF_RR,_ compared to daytime, was observed only in W_NOKID. Indeed, an altered variation of the vagal activity from daytime to nighttime was clearly identified in W_KID^30^.

While during daily hours the two groups of women were characterized by similar CAP, during the nighttime, W_NOKIDS turned to an autonomic “sleep mode”, while W_KID remained in an “active mode”, i.e. they diverted the expected physiological modification of CAP pattern during nighttime^7,35,39^. Of notice, this result seemed to be unrelated with the preschoolers’ age and the number of siblings.

This raises the question whether such modifications may jeopardize these women’s health, as it is well known that a decreased vagal modulation in resting conditions is an independent risk factor for all-cause mortality in several pathological situations, including myocardial infarction, hypertension, heart failure and diabetes^14–17^. On the other hand, it has to be underlined that W_KID showed a normal sympathovagal modulation during daytime with a paradoxical behavior only during the night. This CAP modification could be the results of an adaption modality to maintain a high level of alert aimed at maximizing the survival of both herself and the offspring throughout the 24 hours. Based on the *fight or flight* theory^40^, the sympathetic branch of the autonomic nervous system is more active, causing a concomitant de-activation of the vagal one, in presence of external challenges of the system or emergency situations, to cope with the acute stress^13,41^. As an example, in reaction to the passive orthostatic challenge applied during tilt test, the reduction of the vagal activity aims at maintaining the homeostasis^11,13^. In the case of a woman with the baby sleeping next door, a low level of vagal activity during the nocturnal hours would facilitate a prompt reaction in case of a child’s cry of hunger or a threat.

This is in keeping with the theory that cardiac vagal control may regulate behavioral, cognitive, and emotional responses by inhibiting the central autonomic network^42^.

Nevertheless, some important questions remain open. A prolonged or continuous decreased level of vagal activity could represent for the young mothers an additional risk factor for the development of future cardiovascular events^13,41^. Indeed, a reduced vagal activity directed to the heart during daytime was recently observed in mothers up to 2 years postpartum with symptoms of anxiety and depression^43^.

Moreover, stress and lack of sleep are recognized causes of altered heart rate variability^29,44–46^. Therefore, further longitudinal studies are advocated to verify if the observed vagal hypotonia is only transient, to deepen the relation between the cardiac controls and the mothers’ psychological status, and to eventually include a male population.

The main limitations of the present study are the lack of information about hormonal levels and detailed sleep assessment, which should be evaluated in future studies. However, in the present study, gross sleep parameters, i.e. bedtime, sleeping hours, number of awakenings were similar in both groups. A further limitation is the lack of an accurate evaluation of the perceived stress of the participants. The results of VAS score, although a weak tool given its subjectivity, suggest that this would be an interesting issue to explore. More sophisticated and objective measures of stress should be employed in future studies to add important insights.

Although this is a cross-sectional study of a single 24-h ECG recording, there is strong suggestion that the group of female healthcare professionals engaged in the care of young children seem to have a different cardiac autonomic profile from their peers without such commitment, that is, a reduced nocturnal vagal activity that can favor a prompt response and greater reactivity in case of any need of the offspring.

## Supplementary Information

Supplementary Information.

## Data Availability

Anonymized data are available upon reasonable request to the corresponding author. Potential data exchange will be conducted in accordance with the European legislation about data protection.
