# Supplementary material for: How the first years of motherhood impact the cardiac autonomic profile of female healthcare professionals: a study by heart rate variability analysis
Source: Sci Rep. 2021 Apr 14;11:8161. doi: 10.1038/s41598-021-87596-y (PMC8047021; doi:10.1038/s41598-021-87596-y)
Supplement: Supplementary file 1 — Supplementary Information. [file 41598_2021_87596_MOESM1_ESM.docx]

Supplementary Information

**Results of the ANOVA analysis**

|  | **μ_RR_** | **σ^2^_RR_** | **HF_RR_** |
| --- | --- | --- | --- |
| F-value, DAY vs NIGHT | 105.619 | 2.810 | 13.965 |
| t-value, DAY vs NIGHT | 10.277 | 1.676 | 3.737 |
| p-value, DAY vs NIGHT | <0.001 | 0.100 | <0.001 |
| F-value, K_NOKID vs W_KID | 2.001 | 7.576 | 5.947 |
| t-value, K_NOKID vs W_KID | 1.414 | 2.752 | 2.439 |
| p-value, K_NOKID vs W_KID | 0.164 | 0.008 | 0.018 |
| F-value interactions | 0.476 | 1.995 | 3.475 |
| p-value interactions | 0.494 | 0.164 | 0.068 |
| t-value, DAY vs NIGHT within W_NOKID | 7.755 | 2.184 | 3.961 |
| p-value, DAY vs NIGHT within W_NOKID | <0.001 | 0.034 | <0.001 |
| t-value, DAY vs NIGHT within W_KID | 6.779 | 0.186 | 1.324 |
| p-value, DAY vs NIGHT within W_KID | <0.001 | 0.853 | 0.192 |
| t-value, W_NOKID vs W_KID within DAY | 0.801 | 1.386 | 0.537 |
| p-value, W_NOKID vs W_KID within DAY | 0.425 | 0.169 | 0.593 |
| t-value, W_NOKID vs W_KID within NIGHT | 1.561 | 3.055 | 3.057 |
| p-value, W_NOKID vs W_KID within NIGHT | 0.122 | 0.003 | 0.003 |

RR, RR interval; μ_RR_, mean of RR; σ^2^_RR_, RR variance; HF_RR_, absolute power in high frequency band of the RR; DAY, daytime; NIGHT, nighttime; W_NOKID, women without preschoolers; W_KID, women with preschoolers.
